# The Role of Mesenchymal Stem Cells in Radiation-Induced Lung Fibrosis

**DOI:** 10.3390/ijms20163876

**Published:** 2019-08-08

**Authors:** Michele Zanoni, Michela Cortesi, Alice Zamagni, Anna Tesei

**Affiliations:** Bioscience Laboratory, Istituto Scientifico Romagnolo per lo Studio e la Cura dei Tumori (IRST) IRCCS, 47014 Meldola, Italy

**Keywords:** mesenchymal stem cells (MSCs), radiotherapy, thoracic cancer, lung fibrosis, regenerative medicine

## Abstract

Radiation therapy is one of the most important treatment modalities for thoracic tumors. Despite significant advances in radiation techniques, radiation-induced lung injury (RILI) still occurs in up to 30% of patients undergoing thoracic radiotherapy, and therefore remains the main dose-limiting obstacle. RILI is a potentially lethal clinical complication of radiotherapy that has 2 main stages: an acute stage defined as radiation pneumonitis, and a late stage defined as radiation-induced lung fibrosis. Patients who develop lung fibrosis have a reduced quality of life with progressive and irreversible organ malfunction. Currently, the most effective intervention for the treatment of lung fibrosis is lung transplantation, but the lack of available lungs and transplantation-related complications severely limits the success of this procedure. Over the last few decades, advances have been reported in the use of mesenchymal stem cells (MSCs) for lung tissue repair and regeneration. MSCs not only replace damaged lung epithelial cells but also promote tissue repair through the secretion of anti-inflammatory and anti-fibrotic factors. Here, we present an overview of MSC-based therapy for radiation-induced lung fibrosis, focusing in particular on the molecular mechanisms involved and describing the most recent preclinical and clinical studies carried out in the field.

## 1. Introduction

Radiation therapy is one of the most important treatment modalities for thoracic tumors such as lymphoma and lung, breast and esophageal cancer [1,2]. Despite advances made in radiation techniques enabling dose delivery and distribution directly to the tumor mass with less toxicity to surrounding healthy tissue, radiation-induced lung injury (RILI) still occurs and remains an important dose-limiting issue. Up to 30% of patients undergoing thoracic radiotherapy [3] and more than 40% of patients with lung cancer treated with high-dose radiotherapy [4] are diagnosed with RILI. Numerous factors affect the degree of injury sustained by the lung after irradiation, including age, radiation dose and fractions delivered, and concomitant chemotherapy [5]. The molecular mechanisms responsible for the development of radiation-induced toxicity are still poorly understood and effective treatments to improve patient prognosis are lacking [1,6]. Initially described by Friedenstein as hematopoietic-supportive cells of bone marrow [7], mesenchymal stromal cells were classified as a heterogeneous population of fibroblast-like multipotent cells characterized by the ability to differentiate into tissues of mesodermal origin that exhibit substantial *in vitro* expansion and self-renewal capacity [8]. Currently, mesenchymal stromal cells are isolated from numerous stromal tissues within the body including adipose tissue, placenta, umbilical cord, and dental pulp, and for this reason a more precise characterization of these cells is needed [9]. In fact, the majority of researchers acknowledge that these in vitro isolated cells are represented by a heterogeneous, non-clonal population of stromal cells containing stem cells with different multipotential properties, committed progenitors and differentiated cells [8]. Among these, mesenchymal stem cells (MSCs) represent a subpopulation currently defined by minimal criteria as having plastic adherence properties and the potential for in vitro trilineage differentiation to adipocytes, chondroblasts, and osteoblasts [10]. However, a re-evaluation of this definition is needed. In addition to standard cell surface markers and differential potential ability, more advanced tools should be used to evaluate their transcriptomic, proteomic and secretomic profiles [9,11]. Over the past few decades, interest in the clinical potential of MSCs in regenerative medicine has significantly increased. Furthermore, their relatively easy access, isolation and expansion ex vivo, as well as their ability to repair tissues and modulate immune and stromal cell functions displaying anti-fibrotic activity have aroused the interest of researchers, making MSC-based therapy a promising candidate for many cell-based therapies, including the treatment of RILI.

## 2. Radiation-Induced Lung Injury

Radiation-induced lung fibrosis is characterized by different kinds of lung alterations that lead to progressive and irreversible organ malfunction [12]. The destruction of lung architecture and the accumulation of fibrotic tissue induce modifications in gas exchanges, resulting in increased dyspnea and accumulation of interstitial fluid, and culminating in respiratory failure and death [13]. To date, no effective therapies have been developed or approved for clinical use due to the complexity of this disease. The only drug currently used in a clinical setting is amifostine, a radioprotective agent that scavenges oxidative radicals and accelerates tissue repair [14,15]. However, this compound shows short-term activity and induces important side-effects such as diarrhea, nausea and hypotension [14], thus limiting its clinical use. The most effective intervention for the treatment of RILI is lung transplantation, but the lack of available organs and transplantation-related complications severely limit the success of this procedure [12,16,17]. Hence new and advantageous therapeutic strategies are urgently needed to treat RILI.

### 2.1. Clinical Significance of Lung Fibrosis

The effects of radiation on the lungs are, from a clinical point of view, considered as a continuous and complex process characterized by an initial latent and asymptomatic phase starting immediately after radiation treatment, followed by an acute phase (radiation pneumonitis), and a late phase (radiation-induced pulmonary fibrosis) that normally occurs 1–2 years post-treatment [2]. Although the 2 main phases of radiation pneumonitis and radiation pulmonary fibrosis are interdependent, they are clearly separated in time. The time required for the severity and development of radiation pneumonitis (usually 1–6 months after the end of radiotherapy) are related to different factors such as the volume of lung irradiated, the total dose delivered, the fractionation schedule used and other patient-related factors. Diagnosing radiation pneumonitis is not a simple task, as it must be distinguished from other lung dysfunctions that could appear after radiotherapy such as tumor progression or chronic obstructive pulmonary disease [18]. Radiation pneumonitis is characterized by nonspecific respiratory symptoms, such as cough, low-grade fever, pleuritic and/or chest pain and mild dyspnea [19]. Although there are no tests available to diagnose radiation pneumonitis, the presence of specific features can facilitate its identification. For example, after radiation treatment tissue density increases and other architectural changes occurs such as lung contraction, pleural thickening and volume loss [20]. Chest X-rays (CXRs) and computed tomography (CT) are often already used by clinicians to evaluate local lung changes after radiation therapy [21,22]. Moreover, information on the timing of radiotherapy and on the radiation therapy history of each patient, together with radiographic imaging and transbronchial biopsy referrals, can help to rule out other causes [18]. The treatment of radiation pneumonitis is mainly based on the administration of high-dose oral glucocorticoids (e.g., prednisone) over several weeks [18]. For patients who cannot be treated with steroids, alternative immunosuppressive agents such as azathioprine and cyclosporine [23,24] can be considered. Although patients treated with steroids show good prognosis and a resolution of acute clinical symptoms, the majority go on to develop pulmonary fibrosis [16]. Fibrosis is believed to be the culmination of a series of events triggered by radiation treatments, each contributing to create irreversible damage [25]. Fibrosis is described as a chronic form of lung damage that results in the progressive destruction of lung tissue architecture with the development of a fibrotic “scar”, especially in alveolar areas, leading to reduced gas exchange and chronic respiratory failure [12].

### 2.2. Molecular Mechanisms Involved in RILI

**Figure 1:**
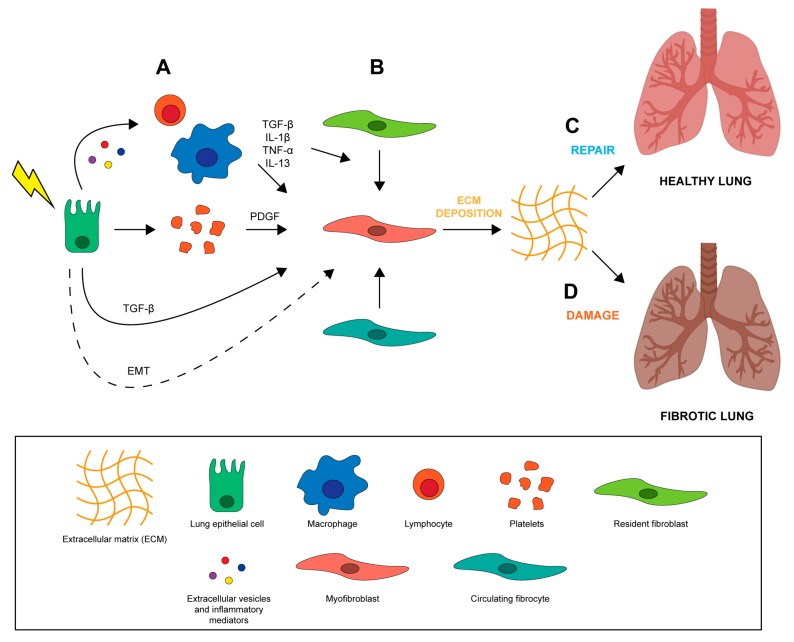
Alterations in normal wound healing processes promote the development of pulmonary fibrosis. (**A**) After radiation injury epithelial cells release inflammatory mediators, triggering platelet aggregation and inflammatory cell recruitment and activation. (**B**) Pro-fibrotic and inflammatory cytokines released by recruited immune cells, such as macrophages and lymphocytes, promote the recruitment and differentiation of resident fibroblasts and circulating fibrocytes into ECM-secreting myofibroblasts. Fibroblasts and myofibroblasts may also originate from epithelial cells that have gone through the EMT process. (**C**) Activated myofibroblasts remodel ECM, actively promoting tissue repair by epithelial and endothelial cells and restoring lung function. (**D**) Disregulation of wound healing process and persisted inflammatory environment promote lung tissue fibrosis [29,30,31,32,33,34,35,36,37,38].

The lung is one of the most sensitive organs to the direct and indirect damage caused by ionizing radiation which frequently overcomes the intrinsic repair capacity of the lung, resulting in a permanent injury. The exact mechanisms involved in the development of RILI are still not known. Radiation disrupts epithelial and endothelial integrity in different areas of lung parenchyma from bronchi to alveolar capillary complexes, leading to a cascade of biomolecular events that modify the microenvironment, thereby inducing inflammation and oxidative stress [26]. One hypothesis is that RILI results from an abnormal healing response. Unlike normal wound healing processes where inflammation is reduced when damaged tissue is repaired, a vicious cycle promoting further inflammation is established during RILI, leading to the accumulation of pro-inflammatory and pro-fibrotic cytokines, fibroblasts, inflammatory cells, and extracellular matrix (ECM) proteins, which may result in lung dysfunction [6,27,28]. Wound healing is a fundamental biological process for the accurate reconstruction of tissue architecture after an injury through the replacement of apoptotic and necrotic cells [29]. It is composed mainly of 4 phases including a coagulation phase, inflammatory phase, proliferative phase, and final remodeling phase [29]. In the early stages of tissue injury, damaged epithelial and endothelial cells release high amounts of inflammatory mediators that promote the activation of a coagulation cascade [30] through platelet activation and aggregation (Figure 1A). This process triggers the formation of clots, provisional ECM and hemostasis [29]. Platelet degranulation improves vasodilation, increasing blood vessel permeability and permitting the recruitment of inflammatory cells (e.g., neutrophils, macrophages and lymphocytes) to the site of injury (Figure 1A). During the initial phases of the wound healing process, activated leucocytes (first neutrophils and then macrophages) eliminate dead cells, debris, and any invading organisms. The leucocytes also secrete cytokines and chemokines such as IL-1β and TNF that amplify inflammatory response and promote the recruitment and proliferation of both endothelial cells and fibroblasts. The subsequent activation of T cells, which secrete IL-13 and TGF-β [31,32], promotes the proliferation of fibroblasts and their conversion into matrix-producing myofibroblasts (Figure 1B) [33]. In the final phase of tissue remodeling, these α-smooth muscle actin-expressing myofibroblasts work by contracting the edges of the wound, pulling them towards the center and eventually closing up the wound. Endothelial and epithelial cells then migrate to the basal layer of provisional ECM to complete the process of regenerating damaged tissue (Figure 1C). Radiation-induced lung fibrosis occurs when the injury is severe and repeated, when there is chronic inflammation, and when the repair mechanisms are dysregulated (Figure 1D). The effect of ionizing radiation on lung tissue is mediated by the generation of reactive oxygen species (ROS) and nitrogen species (RNS) which, coupled with the direct energy transfer of radiation, induce DNA, protein and lipid damage, leading to irreversible cell injury and death [34]. Loss of both epithelial and endothelial cells leads to blood-air barrier dysfunction and increased vascular permeability. In particular, radiation of lung tissue reduces microvessel density and lung perfusion, causing hypoxia and edema [35]. These injuries contribute to establishing a chronic condition of inflammation, stimulating the recruitment of inflammatory cells that are capable of starting the repair process through the elimination of cellular debris and the control of excessive cellular proliferation [4,36]. However, the establishment of chronic inflammation due to irreversible tissue damage is associated with a second wave of cytokine production for up to 8 weeks after irradiation [37]. This second inflammatory wave increases ROS and RNS levels, promoting oxidative DNA damage, TGF-β, IL-1β, IL-6 and TNF-α expression, and hypoxia [36,37]. The accumulation of different cells in the lung such as macrophages, fibroblasts and other immune cells increases oxygen consumption, leading to a hypoxic environment that further stimulates ROS/RNS production and proangiogenic, proinflammatory and profibrinogenic factors [4], perpetuating tissue damage. Furthermore, the different components of the inflammed lung tissue microenvironment secrete vascular endothelial growth factor (VEGF) in an attempt to stimulate the proliferation of the radiation-damaged but still surviving endothelial cells. However, when the accumulated damage is too great they undergo cell death, further worsening the hypoxia and inhibiting the healing process [38].

## 3. Cellular Components Involved in Radiation-Induced Lung Fibrosis

### 3.1. Epithelial Cells

**Figure 2:**
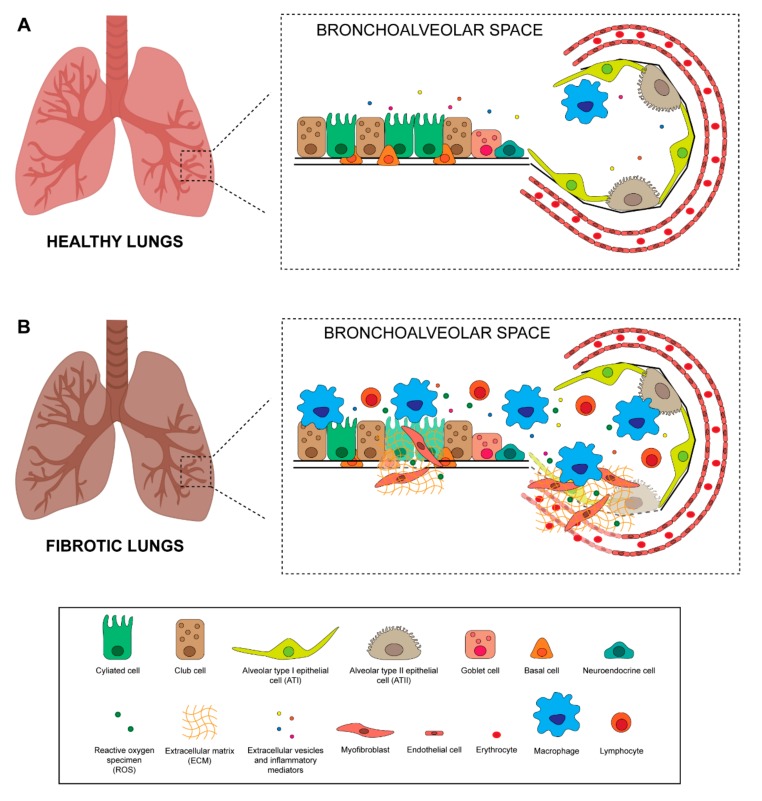
Differences in normal and fibrotic bronchoalveolar tissue. Schematic representation of (**A**) normal bronchoalveolar tissue and (**B**) fibrotic bronchoalveolar tissue [12,14,28,39,40,41,42,43,44,45,46,47,48,49,50,51,52,53,54,55,56,57,58,59,60,61,62,63,64,65,66,67,68,69,70,71,72,73,74,75,76,77,78,79,80,81,82,83].

Many types of cells composing lung tissue are affected by radiation treatment and each can potentially contribute to the development of fibrosis. The airway epithelium comprises multiple cell types constituting a mucosal barrier that mechanically and physically protects it from environmental insults and infections and orchestrates innate and adaptive immune response [39]. The pseudostratified tracheobronchial epithelium is composed of ciliated cells, basal cells, neuroendocrine cells, and secretory club and goblet cells (Figure 2A). Ciliated and secretory cells collaborate to keep the lungs free of inhaled external particles and microorganisms [39]. Goblet and club cells produce and secrete mucous onto the surface of the epithelium. Mucous is a viscous material similar to gel, composed mainly of mucin glycoproteins, glycosaminoglycans, lysozyme, IgA and many cytokines [40]. A high number of basal cells are present under the epithelial layer, probably representing epithelial progenitor cells that replace damaged epithelium, and neuroendocrine cells which play an important role in regulating cell proliferation and differentiation [39]. The number of epithelial basal cells decrease drastically moving deeper into bronchioles and alveoli. Although the bronchiole epithelium is still poorly characterized, it is acknowledged that there are 2 main cell types in the alveoli: alveolar type I (ATI) and type II (ATII) (Figure 2A). ATI cells are very large squamous cells with thin cytoplasmic extensions covering 90–95% of the alveolar surface, and are sensitive to toxic insults and unable to replicate [41]. These cells are involved in gas exchanges and in regulating liquid homeostasis, essentially forming the air-blood barrier within the alveoli [14,41]. ATII cells are granular and cuboidal cells that cover only 5% of the alveolar epithelium. They have 2 main functions, i.e. the synthesis, secretion and regulation of surfactants, a phospholipidic-enriched substances that reduce alveolar surface tension, and proliferation and differentiation into ATI cells, thereby playing a crucial role in the repair and remodeling of the lung following injury [41,42]. Radiation induces oxidative stress through the production of oxygen-free radicals and reactive oxygen specimens (ROS), which damage lung tissue, in particular epithelial and endothelial cells [43] (Figure 2B). It also causes delayed damage to resident lung epithelial cells, resulting in cell apoptosis and in the disruption of the epithelial barrier function [14,28]. Specifically, damaged tissue cells release pro-inflammatory cytokines which, together with altered vasodilation and vascular permeability, promote the recruitment of immune cells, such as macrophages and lymphocytes, to the site of injury [12,44] (Figure 2B). The inflammatory cells recruited in the interstitial space are responsible for the secretion of many cytokines that play a crucial role in initiating and maintaining lung fibrosis, such as IL-6, TNF-α, CTGF, TGF-β and IL-1α [45]. Among these, TGF-β is involved in recruiting fibroblasts and immune cells, in the proliferation and differentiation of myofibroblasts, and in the induction of EMT [46]. Activation of local fibroblasts is needed for the initial step of the fibrotic response but not sufficient for the development of the complete disease [39]. Myofibroblasts, constitutively activated by TGF-β, contribute to oxidative stress by producing hydrogen peroxide that leads to epithelial cell apoptosis [47]. At the site of the injury, myofibroblasts differentiate from recruited local fibroblasts through a platelet-derived growth factor (PDGF), TGF-β and TNF-α secreted by platelets and epithelial cells, respectively [48]. Circulating CXCR4-positive fibrocytes attracted to the lung via the secretion of CXCL12 chemokine by epithelial cells also contribute to the accumulation of myofibroblasts in injured lung tissue [49,50]. ATII cell depletion and senescence after radiation treatment represents another important cause of ineffective alveolar tissue repair. These cells undergo EMT and differentiate directly into ECM-depositing fibroblasts [49], leading to an abnormal deposition of ECM proteins such as collagen type I [51] and alveolar collapse and obliteration by connective tissue. Loss of ATII cells or the development of a senescent phenotype depends on exposure time and on the dose of radiation received [52].

### 3.2. Cells of the Innate Immune Response

Radiation-induced damage in lung tissue leads to the activation of the immune system. Radiation-damaged epithelial and endothelial cells release a variety of chemotactic factors that promote the recruitment of inflammatory myeloid cells at the site of injury involved in chronic inflammation-induced fibrosis [53]. Monocytes and neutrophils respond to chemokines gradients (e.g., chemokine (C–C motif) ligand 2 (CCL2)) and are recruited to damaged tissue where they differentiate into macrophages, phagocyting fibrin clots and cellular debris. Despite the importance of these cells in the wound healing process, the continuous release of toxic mediators (ROS and RNS) and their non rapid elimination further triggers the fibrotic cascade [54]. The macrophage phenotype observed during the peak of the pro-fibrotic immune response usually matches alternative macrophage activation (M2) rather than classic activation (M1) by IFN-γ [53]. M2 macrophages express immunosuppressive molecules such as IL-10 and arginase-1, the latter being especially important for the synthesis of l-proline, an amino acid required for the production of collagen by activated myofibroblasts [55]. M2 macrophages are also involved in the development of T helper type-2 (Th2) lymphocytes which produce pro-fibrotic cytokines and suppress T helper type-1 response (Th1) [56,57]. It remains unclear whether M2 macrophages preferentially infiltrate fibrotic tissue or whether the pro-fibrotic microenvironment drives M2 polarization [58].

### 3.3. Cells of the Adaptive Immune Response

The adaptive immunity response plays an important role in the fibrotic process, in particular Th2 response involving IL-4, IL-5 and IL-13 [59], thus representing a key driver of progressive fibrosis [60]. IL-13 is the predominant regulator of fibrotic tissue remodeling in several models of fibrosis [61] such as idiopathic pulmonary fibrosis (IPF) [62] and radiation-induced fibrosis [63]. This interleukin appears to induce fibrosis by stimulating TGF-β production or by the TGF-β-independent activation of proliferation and synthetic proprieties of fibroblasts, epithelial cells and smooth-muscle cells [64,65]. Studies on transgenic mice overexpressing IL-4 and IL-13 in the lung have confirmed the role of these pro-fibrotic cytokines in directly influencing the activation of myofibroblasts [66]. The pro-fibrotic activity of IL-13 depends on the abundance of the IL-13Rα1 signaling receptor and IL-13Rα2 decoy receptor expressed in target cells, including myofibroblasts [67]. IL-13Rα2 displays greater affinity for IL-13 [68], suppressing IL-13-IL-13Rα1-induced responses and, consequently, pulmonary fibrosis [67,69]. Conversely, when the IL-13Rα2 receptor is absent or lowly expressed, the fibrotic process is enhanced [70]. In addition to Th2 response, the CD4+ IL-17-producing cell subpopulation (Th17), represents an emerging driver of lung fibrosis [54,71,72]. IL-17A produced by Th17 cells is associated with abnormal recruitment of neutrophils, resulting in persistent neutrophilia [73] and an increase in lung fibrosis through the induction of vascular endothelial cell apoptosis [74]. The IL-17A cytokine has been shown to directly regulate both the upstream and downstream phases of fibrosis, inducing the expression of matrix metalloproteinase-1 in primary human cardiac fibroblasts [54,75]. Th17 cells are induced by cytokines abundant in radiation-damaged lung tissue including IL-6, IL-21 and TGF-β [76]. IL-1β and IL-23 are other important upstream factors affecting the Th17 pro-fibrotic response [77]. Regulatory T cells (Tregs) are found in a variety of fibrotic diseases [54] but their role in fibrogenesis is still not fully understood. Foxp3-expressing Tregs produce immunosuppressive cytokines such as IL-10 and TGF-β, suggesting a suppression of the inflammatory cascade that promotes ECM deposition and fibrotic disease [54,78]. Several studies have demonstrated a suppressive role of Tregs in fibrosis models [79,80,81], highlighting a protective role of IL-10 in the progression of the disease [78]. Despite this, few studies have reported a pro-fibrotic role of TGF-β-producing Tregs [82,83]. It is unclear why Tregs show anti-fibrotic activity in some cases and pro-fibrotic activity in others [54], but such behavior is suggestive of an interaction with other T cell population effectors in fibrogenesis.

## 4. Mesenchymal Stem Cells

**Figure 3:**
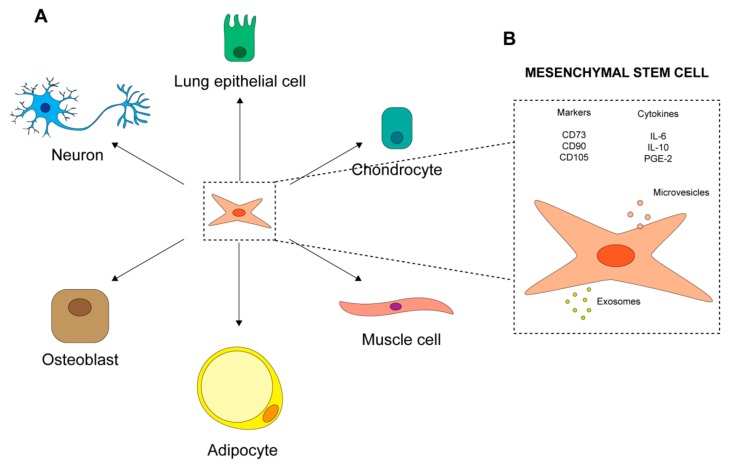
Mesenchymal stem cells (MSCs). (**A**) Multipotency of MSCs. (**B**) MSC markers and the secretome [7,9,10,84,85,86,87,88,89,90,91,92,93,94,95,96,97,98,99,100,101].

Mesenchymal stromal cells were first identified in 1968 [7] as a fibroblastic-like subpopulation of bone marrow cells endowed with clonogenic ability and plastic adherence capacity [7]. As MSCs are represented by heterogeneous cell populations, the International Society for Cellular Therapy (ISCT) defined minimal criteria for their identification [10]. According to these criteria, candidate MSCs must show plastic adherence and demonstrate their multipotentiality by being able to differentiate into adipocytes, chondrocytes and osteocytes (Figure 3A). Additionally, these cells commonly express surface markers such as CD105 (endoglin, SH2), CD73 (ecto-50-nucleotidase) and CD90 (Thy1), but they lack hematopoietic markers CD45, CD19 or CD79, CD14 or CD11b, and HLA-DR [9] (Figure 3B). MSCs are capable of migrating to lesion sites and of differentiating into tissue-specific functional cells (e.g., lung [84], smooth-muscle [85] and neuron cells [86] (Figure 3A). They also promote cell survival and repair of damaged resident cells, thus enhancing the regeneration of injured tissue [87]. Moreover, they exert documented immune-regulatory effects on the immune system, acting on both adaptive and innate responses. MSCs suppress T cells [88], reduce B cell activation and proliferation [89], and actively interact with macrophages, exerting both anti-inflammatory and pro-inflammatory effects [9,90,91]. Furthermore, human MSCs lack immunogenicity and antigen-presenting capacity [92], probably due to the absence of MHC II and T-cell co-stimulatory molecules such as B7-1, B7-2, CD40 and CD40L [93]. Finally, MSCs are highly metabolically active and their secretome gives rise to the same effects commonly described for the cells themselves [94]. MSCs are reported to secrete chemokines [95,96], cytokines [97,98], growth factors and paracrine molecules [99,100]; these last are present in extracellular vesicles, such as exosomes (Figure 3B). Interestingly, MSC paracrine activity, like that of soluble factors, appears to play a key role in repairing injured tissue caused by different pathological conditions [101].

### 4.1. MSC-Based Therapy: From Biological Aspects to Clinical Use

**Figure 4:**
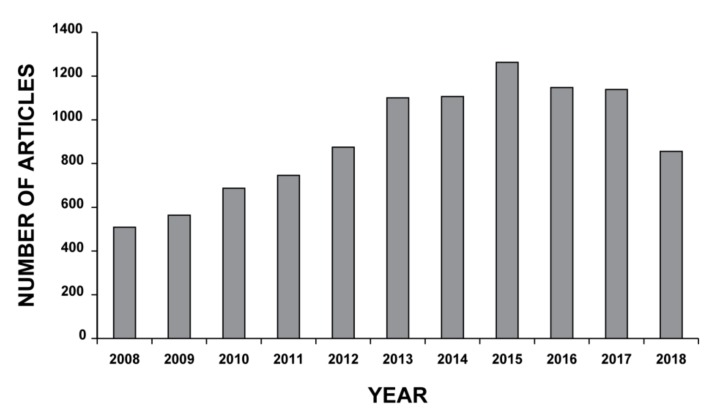
Mesenchymal stem cell-based therapy. Search for article appearing in PUBMED database over the past 10 years using the mesh terms “Mesenchymal Stem Cells” AND “Cell- and Tissue-Based Therapy” in the Advance research builder option.

MSCs display high potential for the treatment of both immunological and non-immunological diseases [102], MSC-based therapy having been successfully used to repair tissues and several organs, including lungs [103]. The last decade has seen an increase in the interest in MSC-based therapy by the scientific community, with more than 800 new research papers published only last year (2018) (Figure 4). Despite this, “stem cell” therapy remains largely unregulated worldwide. Leading countries in the area of regenerative medicine have thus joined forces to establish common regulatory measures to reinforce oversights and guarantee safety in this still rather unexplored scientific territory [104]. In 2017, Japan introduced strict legislation in response to international concerns about the indiscriminate and negligent use of stem cell therapy [105]. Australia, India and the United States have since followed suit, increasing supervision on clinics and implementing more stringent norms to improve safety [106]. Stem cell-based therapy remains less regulated in China and South Korea, with a lack of regulatory guidelines and poor clinical governance in relation to non approved treatments for patients [107,108]. According to the official database of the U.S. National Institutes of Health (https://clinicaltrials.gov), there were 910 clinical studies on MSCs registered as of April 28th, 2019, the majority focusing on MSCs as a potential therapy for nervous system disorders and bone and cartilage diseases. In contrast, only a handful of clinical trials are currently evaluating the regenerative and therapeutic role of MSCs in inflammatory lung disease. Most are phase I or II studies and their aim is to test the feasibility, safety and efficacy of MSC-based therapy, thus indicating that any hopes for its implementation in clinical practice are still a long way off [102]. 

The therapeutic potential of MSCs is a result of unique proprieties such as their ability to migrate to the site of an injury, their secretion of soluble factors required for cell death or survival and for the modulation of the immunological response, their capacity to differentiate into several cell lineages, their low immunogenicity, and the simplicity of their isolation and culture [109,110,111]. After intravenous or intra-arterial infusion MSCs initially remain trapped in the capillary beds of the liver and lungs [112], making these 2 organs easily targetable. However, following an injury, MSCs cross endothelial beds and enter into the site of injury [113,114] through a process called “transendothelial migration” [115]. MSC homing processes are not fully understood but are known to involve several molecules such as chemokine receptors, including CCR2, CCR4, CCR7, CCR10, CXCR5, CXCR6, and CXCR4 [115,116,117,118], adhesion proteins and matrix metalloproteinases (MMPs) [103,119] molecules those are involved also into the well known processes of leukocyte extravasation. Among these, the SDF-1/CXCR4 axis and cytokine receptor CCR2 are important for specific organ homing [118,120]. MSCs migrate through the endothelium, forming bleb-like protrusions similar to those formed by metastatic tumor cells [121,122] and overcoming the basal barrier through the activity of MMPs. In particular, MMP-2 and MT1-MMP have shown to be essential for MSCs migration [123,124]. It was initially thought that the therapeutic proprieties of MSCs derived principally from their ability to differentiate into epithelial-like cells that replace damaged tissue [103]. However, subsequent in vivo studies demonstrated a limited replacement of damaged tissue by differentiated stem cells (about 5%) [103], indicating that the clinical and therapeutic effects derived principally from the endocrine and paracrine mechanisms activated by these cells [6,125] that strongly affected the organ microenvironment and local cellular dynamics [8,102]. MSCs migrating to injured tissue face a complex microenvironment characterized by several chemical and physical stimuli that influence their behavior. Hypoxia and inflammation are common features of injured tissue capable of affecting paracrine effects of MSCs those are principally mediated by VEGF, FGF2, IGF-1, and HGF [94,126]. The biological relevance of these released growth factors was demonstrated in a study by Chang et al. in which only BM-MSCs conditioned medium obtained from hypoxic cultures restored neurological function in a rat model of traumatic brain injury, promoting neurogenesis through VEGF and HGF [127]. Furthermore, hypoxia has been shown to increase the immunomodulatory activity of MSCs by upregulating the indoleamine-2,3-deoxygenase (IDO) enzyme involved in the catabolism of the essential amino acid tryptophan [128]. Non activated MSCs express low levels of IDO, but when stimulated by hypoxia and/or IFN-γ and IDO increases [129]. Indoleamine-2,3-deoxygenase is critical for the immunosuppressive activity of MSCs, inducing T cell anergy [130] and stimulating Tregs [131,132]. It also induces pathways capable of directly or indirectly affecting MSC-mediated immunosuppression. In particular, under inflammatory conditions in the injured microenvironment, IDO, prostaglandin E2 (PGE2) and IL-10 are major effector molecules secreted by MSCs, with immune regulatory function [132,133]. PGE2, synthesized from arachidonic acid by cyclooxygenase enzymes 1 and 2 (COX-1 and COX-2) [134], impacts the proliferation, differentiation and functions of both innate and adaptive immune cells. In particular, PGE2 targets natural killer (NK) cells, monocytes, and the transitional processes of differentiation of macrophages and dendritic cells (DCs) [135,136,137]. IL-10 has well known anti-inflammatory properties because of its induction of immune tolerance [138,139]. It suppresses the activity of macrophages [140], neutrophils [138] and DCs [141], inhibits Th1 response [142], and promotes the expression of anti-inflammatory molecules such as TNF-α, IL-1 antagonists [143] and protease inhibitors [144]. IL-10 inhibits the secretion of DC inflammatory cytokines by suppressing the expression of MHCII complex and B7 on their surface [141]. It also suppresses CD28 expression in T cells, inducing anergy [97,139]. However, IL-10 is also the most widely disputed cytokine, as there are contradictory results on its secretion by MSCs [97,145,146]. 

### 4.2. Radiation Affects MSC Biology

In adult tissues, cells are hierarchically organized and only a small proportion are endowed with self-renewal ability and multi-potential properties which are responsible for the maintenance of tissue homeostasis, balancing stem cell self-renewal and cell differentiation [147,148]. These remarkable functions are accomplished by adult stem cells resident in specific niches in a quiescent status from which they awake under proper stimuli, acquiring the proliferative potential necessary for tissue regeneration [147]. MSCs are a heterogeneous population of differentiated cells, committed-progenitors and stem cells with different multi-potential proprieties [8]. Their role in tissue repair, directly replacing damaged cells, and indirectly modulating immune and stromal cells functions is substantially affected by exposure to both low and high radiation doses [149]. Normal tissue, in particular bone marrow, is particularly sensitive to radiation therapy, with notable differences between differentiated somatic cells and stem cells [150,151]. Ionizing radiation affects DNA structures directly by inducing DNA breaks in 30–40% of lesions, and indirectly by generating ROS which, in turn, damages DNA molecules (60–70% of lesions) [152,153]. Based on the energy delivered, radiation treatment causes different forms of DNA damage [154]. Among the most severe are DNA double-strand breaks (DSB) that activate an intricate signaling network, resulting in a massive accumulation of factors amplifying the damage signal and in the consequent recruitment of key components of DNA damage response mechanisms [155]. Depending on the extent of DNA damage, cells may completely repair the lesion and survive or result permanently blocked and undergo apoptosis, senescence or necrosis [148,150]. MSC response to radiation has been investigated in both mouse and human tissue, studies highlighting a higher in vitro survival and radiation resistance of MSCs compared to other bone marrow-derived stem cells (e.g., hematopoietic stem cells) [150] and embryonic stem cells [156]. In particular, radiation resistance is increased in MSCs exposed to low-dose fractionated radiation treatments [157] which affect the expression of genes involved in stress response, DNA repair and replication [158] and cell proliferation [159]. Furthermore, the radiation resistance properties of MSCs are more pronounced in hypoxic conditions due to the increased activity of DNA repair mechanisms [160]. They have also been confirmed in vivo in MSCs extracted from porcine mandibular bones exposed to 18 Gy dose of radiation, which retained their ability to proliferate and differentiate into osteogenic and adipogenic lineages [161]. Potential mechanisms underlying the radioresistance of MSCs include the activation of multiple downstream DNA damage sensing (e.g., ATM) and repair proteins, as well as the induction of proteins involved in cell cycle regulation such as p53 and p21 [162]. Experimental studies have shown that MSCs are endowed with a highly efficient repair system for DSB lesions, and are capable of activating both NHEJ and HR pathways [162,163,164]. Radioresistant MSCs also display a high expression of anti-apoptotic factors such as BCL-2 and BCL-XL, and low levels of pro-apoptotic factors such as Puma [164,165]. In fact, MSCs mainly undergo senescence [166], even after low-dose radiation [148], and only sporadically apoptosis and autophagy [151,167]. Senescence in MSCs appears to be induced by the retinoblastoma protein pRb and by p53 [149,168] acting through a reduction in the differentiation potential of MSCs [168]. Finally, senescent MSCs secrete a high number of molecules affecting surrounding cells that have a potentially positive or negative effect on the body [148]. The large number of cytokines, extracellular vesicles and growth factors affect the immune system and surrounding cellular behavior, thus impacting tissue physiology. Further studies on MSC radiobiology will help to shed light on the clinical impact of MSC-based therapy in lung fibrosis.

### 4.3. MSC-Based Therapy for RILI

**Figure 5:**
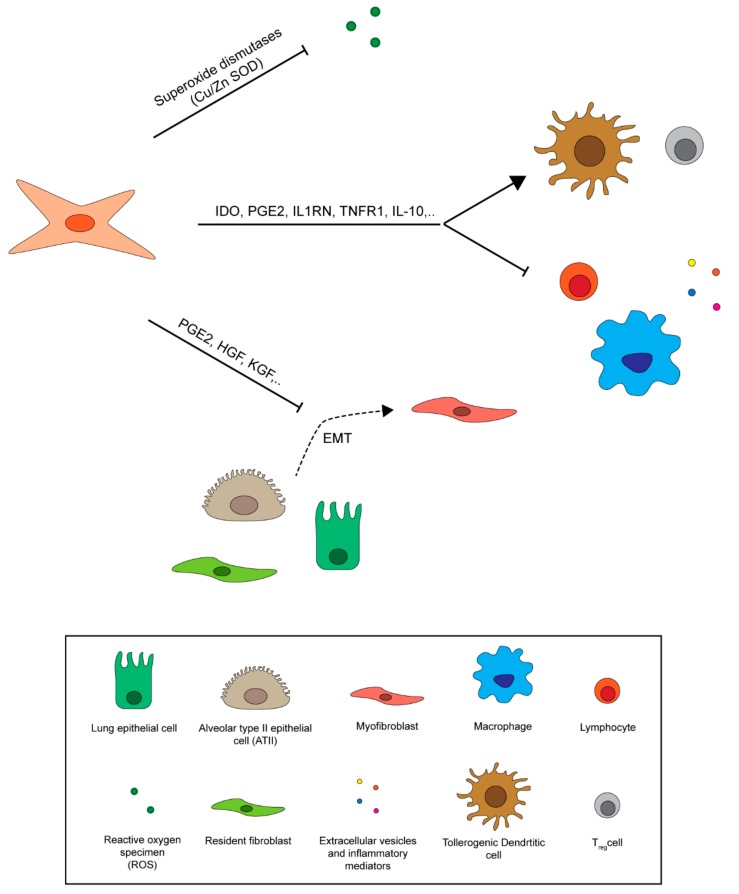
Mesenchymal stem cell regulation of the lung fibrotic microenvironment. MSCs protect radiation-injured lungs against ROS secreting superoxide dismutase enzymes and reduce inflammatory signaling, inhibiting immune cell activation by the release of immunosuppressive cytokines. They also limit fibrotic response by reducing myofibroblast differentiation from epithelial cells and fibroblasts and ECM deposition [6,15,28,46,114,169,170,171,172,173,174,175,176,177,178,179,180,181,182,183,184,185,186,187,188,189,190,191,192,193,194,195,196,197,198,199,200].

The therapeutic potential of MSCs in the treatment of RILI derives mainly from their ability to reach the injured lung tissue after systemic administration and to secrete factors that act on the lung microenvironment [169,170] (Figure 5). Several studies have reported that injured lungs retain MSCs (i.e., BM-MSCs), enabling them migrate to lung airways where they differentiate into epithelial cells [114,171]. In particular, in vitro experiments of MSCs co-cultured with irradiated lung biopsies have confirmed their capacity to differentiate into epithelial cells [172]. Despite this, MSCs are rapidly removed from tissue after transplantation, suggesting that their regenerative effects are mainly due to their secretome [173,174]. ROS generation, the release of pro-inflammatory cytokines (e.g., IL-1α, IL-1β, IL-6) and pro-fibrotic mediators (e.g., TGF-β), and the abnormal deposition of ECM are all hallmarks of RILI [15,28,175]. MSCs reduce oxidative stress and ROS levels through the production of antioxidant enzymes (e.g., superoxide dismutase 1 (SOD1) and 3 (SOD3), manganese superoxide dismutase (MnSOD)), protecting lungs from radiation-induced endothelial damage [176]. SOD1 catalyzes the conversion of superoxide radical into oxygen and hydrogen peroxide, thus protecting cells during the initial phase of RILI [177,178]. MSCs engineered with SOD3 or MnSOD genes show increased anti-fibrotic effects in irradiated lungs with respect to non-modified MSCs [179,180]. Another potent ROS scavenger involved in the anti-fibrotic effects of MSCs is thioredoxin-1 (Trx-1), which also has immune control functions [6,181]. This molecule has been shown to prolong survival in animal experiments, probably decreasing the inflammatory response to radiation by reducing inflammatory cell migration [181]. It has been demonstrated that MSCs directly affect myofibroblast accumulation and reduce myofibroblast differentiation after radiation by acting on the Wnt-pathway [15,182]. TGF-β is a key factor in the EMT process and myofibroblast differentiation [46]. It has been shown that human adipose tissue-derived MSCs (AD-MSCs) downregulate TGF-β1 and TNF-α levels by secreting PGE2 and HGF [183]. PGE2 inhibits TGF-β1-induced proliferation of fibroblasts and collagen production, inducing myofibroblast apoptosis by increasing PTEN activity [184,185,186,187,188]. HGF secreted by MSCs reduces EMT in ATII cells through c-Met, thus inducing myofibroblast apoptosis in an MMP-dependent manner [189,190]. MSCs modified to overexpress HGF are capable of reducing radiation-induced lung fibrosis [191]. Keratinocyte growth factor (KGF) is another important molecule involved in pulmonary epithelial repair, as shown in a study in which BM-MSCs modified to express KGF proved effective against bleomycin-induced pulmonary fibrosis in mice [192]. MSCs paracrine release of interleukin 1 receptor antagonist (IL1RN) and soluble TNF receptor 1 appears to act as a competitive inhibitor of IL-1α and IL-1β, blocking their activity and also that of TNF-α in lung [193,194]. Immunosuppressive cytokines secreted by activated MSCs minimize lung damage caused by abnormal inflammation by acting on functions and proliferation of macrophages, neutrophils, T cells, NK cells and DCs [195]. In the treatment of RILI with MSCs, 2 important factors to take into account are the time window between radiation and MSC administration, and the source of MSCs as these influence efficacy and behavior [6]. An interesting study by Yan et al. analyzed the response of MSCs transplanted at different time points after lung irradiation, with the authors reporting that MSCs injected immediately after radiation treatment reduced lung injury and increased tissue repair, whereas MSCs injected a few months after irradiation contributed to the development of lung fibrosis [196]. To date, the majority of studies evaluating the use of MSCs for RILI have been conducted on BM-MSCs encountering problems in preclinical and clinical practice such as painful cell harvesting and low in vitro expansion capability compared to other MSCs [197]. AD-MSCs and UC-MSCs, on the other hand, show several advantages over MSCs including low immunogenicity and easy in vitro propagation. Moreover, they do not require invasive harvesting procedures, an important ethical issue in stem cell-based therapy [198]. In particular, UC-MSCs from humans proved effective in a rat model of radiation pneumonitis [199], while AD-MSCs infused into a mouse model of acute radiation pneumonitis prolonged survival and decreased levels of inflammatory and fibrotic mediators [200].

### 4.4. Clinical Studies on MSC-Based Therapy for RILI

**Figure 6:**
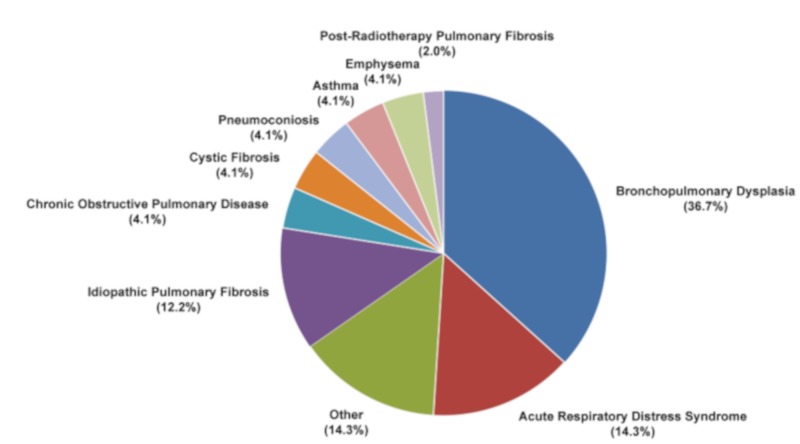
Mesenchymal stem cell-based clinical trials in lung diseases. Search for clinical studies reported in https://clinicaltrials.gov database using “Mesenchymal Stem Cells” and “Lung Disease” as keywords for the search.

Despite in vitro and in vivo evidence of the effectiveness of MSC-based therapy for RILI, clinical trials are still lacking [201]. Up to now the majority of MSC-based lung studies have focused on chronic inflammatory diseases, including bronchopulmonary dysplasia (about 36.7%), acute respiratory distress syndrome (14.3%) and idiopathic pulmonary fibrosis (IPF) (12.2%) (Figure 6), many with the safety and effectiveness of MSC-based therapy as primary endpoints. A phase I clinical trial on IPF patients treated with endobronchial administration of autologous AD-MSCs reported promising safety results together with improved quality of life and overall progression-free survival [202]. In another study, 11 patients with breast cancer or lymphogranulomatosis treated with autologous MSCs in combination with standard therapies showed stabilization of the response to radiation, therefore recording no adverse changes in inflammatory and immune state [203]. There is currently only one active phase I study on the clinical use of UC-MSCs in post-radiotherapy pulmonary fibrosis (https://clinicaltrials.gov: NCT02277145), indicating that there is still a long way to go before MSCs-based therapy can be implemented in clinical practice for RILI (Figure 6).

## 5. Conclusions

Lung pneumonitis and fibrosis remain the major clinical complications of radiotherapy in patients with thoracic tumors, as they reduce the chances of survival and quality of life. The lack of effective therapeutic strategies and the recent advances in MSC-based therapy have induced researchers to investigate the role of MSCs in the treatment of RILI, but several concerns have been raised about their safety. For example, the lack of standardized methods for MSC harvesting and the little or no information available on the optimal dosage, timing and route of administration make it difficult to envisage using MSC-based therapy in clinical practice in the near future. Numerous other questions remain to be answered: What is the best source of MSCs? The best administration route? Are MSCs completely safe? How can MSCs be modified to increase their efficacy and safety? These and many other issues must be addressed before moving forward.

## Abbreviations

AD-MSCs	Adipose tissue-derived mesenchymal stem cells
ATI	Alveolar type I
ATII	Alveolar type II
BM-MSCs	Bone marrow-derived mesenchymal stem cells
CCL2	Chemokine (C-C motif) ligand 2
CCR10	C-C chemokine receptor type 10
CCR2	C-C chemokine receptor type 2
CCR4	C-C chemokine receptor type 4
CCR7	C-C chemokine receptor type 7
COX-1	Cyclooxygenase-1
COX-2	Cyclooxygenase-2
CT	Computer tomography
CTGF	Connective tissue growth factor
CXCL12	C-X-C motif chemokine 12
CXCR4	C-X-C chemokine receptor type 4
CXCR5	C-X-C chemokine receptor type 5
CXCR6	C-X-C chemokine receptor type 6
CXR	Chest X-ray
DCs	Dendritic cells
ECM	Extracellular matrix
FGF2	Fibroblast growth factor 2
HGF	Hepatocyte growth factor
HLA-DR	Human leukocyte antigen - DR isotype
IDO	Indoleamine-2,3-deoxygenase
IFN-γ	Interferon gamma
IGF-1	Insulin-like growth factor 1
IL-10	Interleukin 10
IL-13	Interleukin 13
IL-1α	Interleukin 1 aplha
IL-1β	Interleukin 1 beta
IL-1β	Interleukin 1 beta
IL-21	Interleukin 21
IL-23	Interleukin 23
IL-4	Interleukin 4
IL-5	Interleukin 5
IL-6	Interleukin 6
IPF	Idiopathic pulmonary fibrosis
ISCT	International Society for Cellular Therapy
KGF	Keratinocyte growth factor
MHC II	Major histocompatibility complex II
MMPs	Matrix metalloproteinase
MnSOD	Manganese superoxide dismutase
MSCs	Mesenchymal stem cells
PDGF	Platelet-derived growth factor
PGE2	Prostaglandin E2
PTEN	Phosphatase and tensin homolog HGF
RILI	Radiation-induced lung injury
RNS	Reactive nitrogen species
ROS	Reactive oxygen species
SDF-1	Stromal-Derived Factor 1
SOD1	Superoxide dismutase 1
SOD3	Superoxide dismutase 3
Tregs	Regulatory T cells
TGF-β	Transforming growth factor beta
TNF	Tumor necrosis factor
TNF-α	Tumor necrosis factor alpha
Trx-1	Thioredoxin-1
UC-MSCs	Umbilical cord-derived mesenchymal stem cells
VEGF	Vascular endothelial growth factor
